# NT-proBNP Best Predictor of Cardiovascular Events and Cardiovascular Mortality in Secondary Prevention in Very Old Age: The Leiden 85-Plus Study

**DOI:** 10.1371/journal.pone.0081400

**Published:** 2013-11-21

**Authors:** Petra G. van Peet, Yvonne M. Drewes, Anton J. M. de Craen, Jacobijn Gussekloo, Wouter de Ruijter

**Affiliations:** 1 Department of Public Health and Primary Care, Leiden University Medical Center, Leiden, The Netherlands; 2 Department of Gerontology and Geriatrics, Leiden University Medical Center, Leiden, The Netherlands; University of Louisville, United States of America

## Abstract

**Background:**

In the aging population cardiovascular disease (CVD) is highly prevalent. Identification of very old persons at high risk of recurrent CVD is difficult, since traditional risk markers loose predictive value with age.

**Methods:**

In a population-based sample of 282 85-year old participants with established CVD from the Leiden 85-plus Study, we studied predictive values of traditional cardiovascular risk markers, a history of major CVD (myocardial infarction, stroke or arterial surgery), and new cardiovascular biomarkers (estimated glomerular filtration rate (MDRD), C-reactive protein (CRP), homocysteine and N-terminal pro B-type natriuretic peptide (NT-proBNP)) regarding 5-year risk of recurrent cardiovascular events and mortality (composite endpoint).

**Results:**

During complete 5-year follow-up 157 (56%) participants died. 109 (39%) had a cardiovascular event or died from cardiovascular causes. Individually related to the composite endpoint were: a history of major CVD (HR 1.5 (95%CI 1.03-2.3)), CRP (HR 1.3 (95%CI 1.03-1.5)), homocysteine (HR 1.4 (95%CI 1.2-2.6)) and NT-proBNP (HR 1.7 (95%CI 1.4-2.1)). A prediction model including all traditional risk markers yielded a C-statistic of 0.59 (95%CI 0.52-0.66). Of all five new markers only addition of NT-proBNP improved the C-statistic (0.67 (95%CI 0.61-0.74, p=0.023)). The categoryless net reclassification improvement for NT-proBNP was 39% (p=0.001), for a history of major CVD 27.2% (p=0.03) and for homocysteine 24.7% (p=0.04).

**Conclusions:**

Among very old subjects with established CVD, NT-proBNP was the strongest risk marker for cardiovascular events and cardiovascular mortality. When estimating risk in secondary prevention in very old age, use of NT-proBNP should be considered.

## Introduction

In the aging population, cardiovascular disease (CVD) is highly prevalent and remains a leading cause of death [1,2]. Persons with previous CVD are known to be at high risk of recurrent CVD [3–7]. However, even though secondary preventive treatment is effective in very old age [8–11], treatment at this age is often far from optimal [12–15] and drug adherence is poor [16]. Identifying patients at highest risk of recurrent events can help clinicians to select those very old patients that might benefit most from intensified preventive lifestyle measures and drug treatment [17]. 

In secondary prevention, traditional risk markers seem to have less predictive value [18]. However, data on their actual value in secondary prevention in very old age are scarce. In search of improvement of risk stratification, some studies found additional predictive value by including information on various degrees of previous CVD [4,19,20]. Many studies have evaluated the additional predictive value of markers of renal dysfunction (MDRD, albuminuria or cystatin C), inflammation (C-reactive protein (CRP)), oxidative stress (homocysteine) or myocardial wall stress (N-terminal pro B-type natriuretic peptide (NT-proBNP)), but nearly all in a primary preventive setting [21–25] and especially aimed at improving prediction in those with intermediate risk. Although some studies have shown incremental predictive value of new biomarkers in populations with established CVD [20,23,26–31], their predictive value in a secondary preventive setting in very old age remains unknown.

We hypothesized that the predictive value of traditional risk markers in secondary prevention in very old age is limited and that addition of information on the history of CVD or new biomarkers (MDRD, CRP, homocysteine and NT-proBNP) might have incremental value for predicting cardiovascular events and cardiovascular mortality. 

## Methods

### Study design and participants

The Leiden 85-plus Study is a prospective population-based study in 85-year-old inhabitants of the city of Leiden in the Netherlands [32]. In brief, between September 1997 and September 1999, 705 people from the 1912-14 birth cohort living in the city of Leiden reached the age of 85 years and were eligible to participate. No exclusion criteria were used. From the 705 people who were eligible at age 85, 92 refused participation and 14 died before enrolment. A total of 599 (87%) people gave informed consent and were enrolled. 

At baseline and yearly up to age 90 years participants were visited at their place of residence to obtain extensive data on health and functioning; blood samples and an ECG were taken. Medical history and CVD status were obtained from the medical records of the participant’s physician. Pharmacists provided information on all medication used by the participants. 

The Medical Ethics Committee of the Leiden University Medical Center approved the study. 

Written informed consent was obtained from all participants. The protocol adhered to the principles of the Declaration of Helsinki.

### Baseline Assessment of Risk Markers

#### Traditional risk markers

Blood pressure was measured on two occasions with a mean interval of two weeks. Systolic blood pressure was recorded at the onset of Korotkoff phase I. The mean of the measured systolic values was used for analyses. 

Serum concentrations of total cholesterol and high-density lipoprotein were analyzed on fully automated computerised analyzers (Hitachi 747 and 911; Hitachi, Tokyo, Japan).

Diabetes mellitus was considered present when listed in the medical records of the participant’s physician, when non-fasting glucose concentrations were ≥11.0 mmol/l, or when a participant was taking antidiabetic medication according to the pharmacist’s records.

All participants were interviewed about present smoking habits and were considered as smokers if they were a current smoker of cigarettes, cigars or a pipe. 

#### Nature of the history of cardiovascular disease

For each participant, the primary care physician was interviewed about the CVD history using a standardized questionnaire including questions on present and past cardiovascular pathologies, including myocardial infarction (MI), stroke, surgery for arterial disease, angina, transient ischemic attack (TIA), intermittent claudication and heart failure. The diagnosis of heart failure was based on information as obtained from the participant’s general practitioner or nursing home physician regarding previous events and prevalent disease at entry of the study at age 85 years. An ECG was recorded at baseline and transmitted to the ECG Core laboratory in the Glasgow Royal Infirmary (Scotland, UK) for automated Minnesota Coding [33]. Presence of a MI on the ECG was defined as the presence of Minnesota Code 1-1 or 1-2 (excluding 1-2-8).

Major CVD was considered present if there was a history of MI, stroke, or arterial surgery or if there was a MI on the ECG at baseline [19]. Minor CVD was considered present if there was a history of angina, TIA, intermittent claudication or heart failure.

#### New risk markers

MDRD was calculated as follows: MDRD (ml/min/1,73 m2) = 186 * (serum creatinine (umol/l) / 88, 4)^-1154^, * age (in years) ^–0,203^ * 0,742(for females).

Plasma concentrations of CRP were measured using a fully automated Hitachi 747 analyzer (Hitachi, Tokyo, Japan; detection limit 1 mg/l; coefficient of variation <5%).

Concentrations of homocysteine were measured in plasma samples with a fluorescence polarisation immunoassay after reduction to the free form with an IMx analyzer (Abbott, Abbott Park, IL, USA; coefficient of variation 2.2-2.5%).

NT-proBNP was determined with a chemiluminescent enzyme immunoassay (CLEIA) procedure (Roche, Switzerland) and was carried out on a PATHFAST (Mitsubishi Chemical Medience Corporation, Tokyo.) Detection range was 15 - 30 000 pg/mL and the coefficient of variation was < 5%.

#### Composite endpoint

The composite endpoint ‘cardiovascular morbidity and cardiovascular mortality’ was defined as incident fatal and non-fatal myocardial infarction, incident fatal and non-fatal stroke or any other cardiovascular mortality, whichever came first.

Up to age 90 years all incident fatal and non-fatal MIs were annually registered using data from the primary care physician, ECGs and death registration forms. Incident MI on the ECG was defined as the appearance of Minnesota Code 1-1 or 1-2, or Minnesota Code 1-3 in combination with the first appearance of Minnesota Code 5-x in the same myocardial area [33]. A fatal incident MI was categorised by cause of death codes I21-I23 (ICD 10). Information on incident stroke was collected annually from the primary care physician up to age 90 years. A fatal incident stroke was categorised by cause of death codes I61-I69 (ICD10). All participants were followed for mortality until age 90 years. Date and cause of death were obtained from civic and national registries. Causes of death were divided into cardiovascular causes (ICD-10 codes I00-I99) and non-cardiovascular causes (all other ICD-10 codes). Assignment of cause of death was done blinded for baseline and follow-up study data.

### Statistical analysis

Variables that were unevenly distributed were log transformed.

For all traditional risk markers, a history of major CVD, as well as, the four new biomarkers, hazard ratios (HRs) and corresponding 95% confidence intervals (CIs) for cardiovascular morbidity and mortality were calculated univariate and multivariable, using Cox proportional hazards models, all adjusted for sex. Continuous variables were entered into the model per SD increase.

We constructed prediction models with the traditional risk markers (reference model), and with combinations of the traditional and new risk markers. All biomarkers were entered in the models as continuous variables. For each participant the linear predictor score (X-β) was calculated, using Cox proportional hazard models, all adjusted for sex. Using the continuous predicted risks from each model, C-statistics and receiver operating characteristic (ROC) curves with p-values (level of significance 5%) and 95% CIs were calculated.

We compared the tertiles of predicted risk of the traditional risk marker model and of new models with the observed 5-year incidence of the endpoint, using Kaplan-Meier plots adjusted for competing risks [34] and the log rank test.

Since clinically meaningful risk categories in secondary prevention are not defined, we also calculated the categoryless Net Reclassification Index (NRI), comparing new models to the reference model [35,36]. 

We estimated the integrated discrimination improvement (IDI) [37], a quantification of the difference in sensitivities and ‘one minus specificities’ between new models and the reference model over all possible cut-offs. In addition, we calculated the relative integrated discrimination improvement (rIDI) by dividing the integrated discrimination improvement by the discrimination of the reference model [38].

As a sensitivity analysis regarding the prognostic value of NT-proBNP we repeated all analyses with exclusion of participants with a history of heart failure.

To investigate the validity of our results, we repeated the calculations of C-statistics using cross validated X-beta values obtained by the ‘jack-knife’ method [39]. This was done for the prediction models with 1/ all traditional risk markers, 2/ all traditional risk markers plus NT-proBNP, and 3/ all traditional risk markers plus all five new markers.

Data analysis was performed using SPSS 20 for Windows (SPSS Inc., Chicago, IL, USA). Differences in C-statistics were analysed using Stata/IC 10.0.

## Results

### Baseline characteristics

Of the 599 participants, all aged 85 years at baseline, 300 (50%) had established CVD. Of these 300 participants, for 282 (94%; 109 males, 173 females) all clinical and laboratory data were available and they were included in our analyses. Of these 282 participants 55% had a history of major CVD (32% with MI or MI on the baseline ECG, 19% with stroke and 13% with arterial surgery) and 45% had a history of minor CVD (Table 1). Median NT-proBNP levels were 649 pg/ml (IQR 231-1477) in the subgroup with major CVD, and 405 pg/ml (174-1196) in the subgroup with minor CVD (p=0.035).

**Table 1:** Baseline cardiovascular characteristics of participants with a history of cardiovascular disease at age 85 years (N=282).

		N (%), mean (SD) or median (IQR)**^*a*^**
Traditional risk markers		
	Men	109 (39%)
	Systolic blood pressure, mm Hg	155 (19)
	Total chol, mmol/L	5.7 (1.2)
	HDL chol, mmol/L	1.3 (0.40)
	Diabetes	49 (17%)
	Current smoking	47 (17%)
Nature of cardiovascular disease history**^*c*^**		
	Myocardial infarction	90 (32%)
	Stroke	52 (19%)
	Arterial surgery	37 (13%)
	Angina	105 (38%)
	Transient ischemic attack	72 (26%)
	Intermittent claudication	34 (12%)
	Heart failure	68 (24%)
	Major CVD	155 (55%)
	Minor CVD	127 (45%)
Cardiovascular medication		
	Aspirin or oral anticoagulant	127 (45%)
	Antihypertensive medication**^*d*^**	202 (72%)
	Lipid lowering drug	5 (2%)
New risk markers		
	MDRD, ml/min	57 (15)
	CRP, mg/L	5 (2-9)
	HCY, umol/L	14 (11-17)
	NT-proBNP, pg/ml	495 (198-1314)

*a* data presented as N (%) for categorical variables, mean (SD) for normally distributed or median (IQR) for skewed continuous variables,

*b* assessed only in participants with MMSE >18,

*c* according to treating primary care physician

*d* β-blockers, ACE inhibitors, diuretics and/or Calcium channel blockers

### Incidence of composite endpoint

Of the 282 participants, 157 (56%) died during the 5-year follow-up, of whom 67 (43%) died from cardiovascular causes. In total 109 (39%) participants experienced the endpoint: 43 (39%) participants experienced a fatal or non-fatal MI, 46 (42%) a fatal or non-fatal stroke, and 20 (18%) died of other cardiovascular causes. 

### Univariate and multivariable analyses

Table 2 presents the univariate and multivariable hazard ratios (HRs) for the endpoint for the traditional risk markers, for a history of major CVD, and for the four new biomarkers. In univariate analyses (all adjusted for sex) of the traditional risk markers, current smoking (HR 1.9 (95% CI 1.2-3.0)) was associated with a higher risk. A history of major CVD yielded a HR of 1.5 (95% CI 1.03-2.3). Of the new biomarkers CRP (HR 1.3 (95% CI 1.03-1.5)), homocysteine (HR 1.4 (95% CI 1.1-1.6)) and NT-proBNP (HR 1.7 (95% CI 1.4-2.1)) were associated with a higher risk, whereas MDRD was not (HR 0.83 (95% CI 0.68-1.01)). 

**Figure 1:**
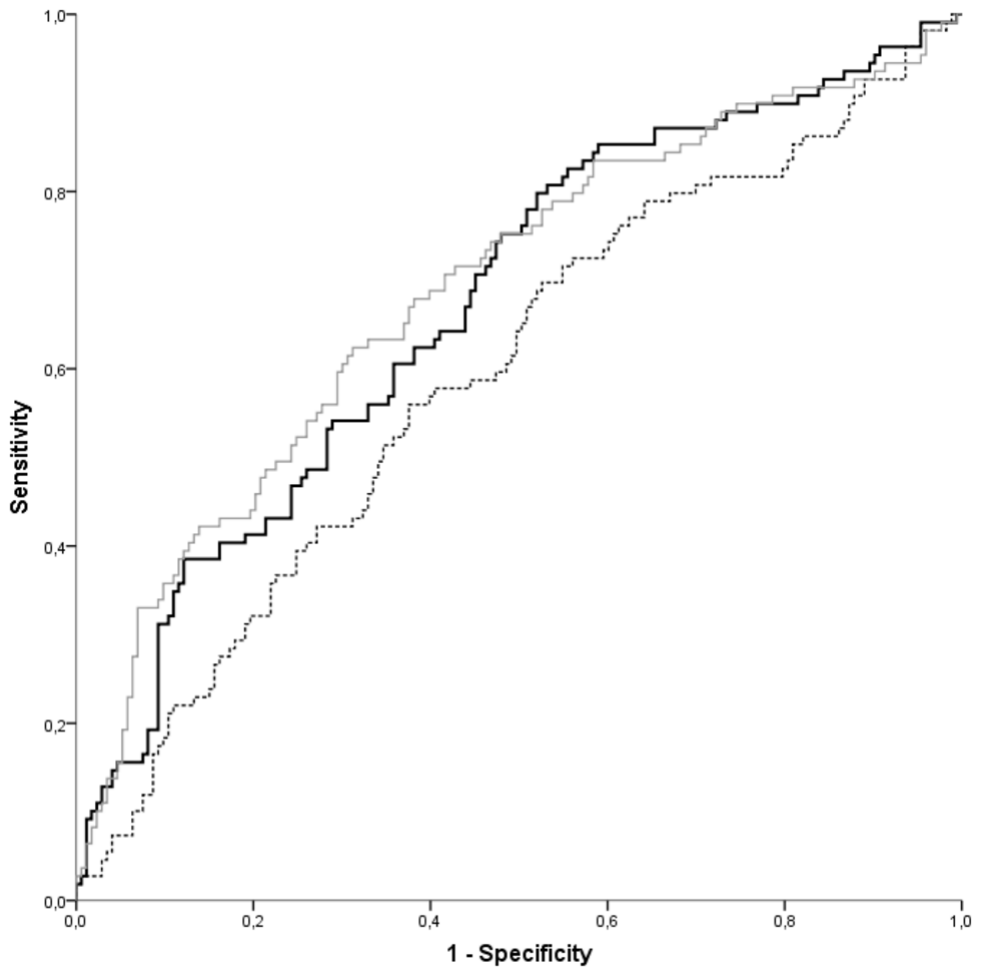
ROC curves for cardiovascular morbidity and mortality. ROC curves for cardiovascular morbidity and mortality of three models: traditional risk markers (dotted line), traditional risk markers plus NT-proBNP (black line, p=0.023), and traditional risk markers plus all five new markers (a history of major CVD, MDRD, CRP, homocysteine and NT-proBNP) (grey line, p=0.0067) (N=282).

**Table 2:** Univariate (adjusted for sex) and multivariable hazard ratios (HR) for five-year cardiovascular morbidity and cardiovascular mortality^***a***^, depending on traditional and new risk markers, in participants with a history of cardiovascular disease (N=282).

		HR (95% confidence interval) for cardiovascular morbidity and mortality**^*a*^**							
		Univariate	Multivariable						
			All traditional	All trad.	All trad.	All trad.	All trad.	All trad.	All trad.
				+ major CVD	+ MDRD (SD)	+ CRP (SD)	+ HCY**^*b*^** (SD)	+ NT-proBNP (SD)	+all new
Traditional risk markers									
	Men	1.4 (0.96-2.0)	1.3 (0.85-2.0)	1.2 (0.79-1.9)	1.4 (0.93-2.2)	1.3 (0.86-2.0)	1.3 (0.85-2.0)	1.3 (0.83-1.9)	1.2 (0.74-1.8)
	RR (∆10 mmHg)	0.95 (0.86-1.1)	0.93 (0.84-1.0)	0.93 (0.84-1.0)	0.93 (0.84-1.0)	0.92 (0.83-1.0)	0.95 (0.85-1.1)	0.96 (0.86-1.1)	0.96 (0.87-1.1)
	Total chol (SD)	1.1 (0.91-1.4)	1.1 (0.92-1.4)	1.1 (0.91-1.4)	1.1 (0.91-1.4)	1.2 (0.94-1.4)	1.1 (0.91-1.4)	1.2 (0.99-1.5)	1.2 (0.98-1.5)
	HDL chol (SD)	1.0 (0.83-1.3)	1.0 (0.82-1.2)	1.0 (0.3-1.3)	1.1 (0.87-1.3)	1.1 (0.87-1.3)	1.1 (0.88-1.3)	1.1 (0.86-1.3)	1.1 (0.92-1.4)
	DM	1.1 (0.66-1.8)	1.1 (0.70-1.9)	1.1 (0.68-1.9)	1.1 (0.67-1.8)	1.1 (0.67-1.8)	1.2 (0.70-1.9)	1.0 (0.62-1.7)	1.0 (0.62-1.7)
	Current smoking	1.9 (1.2-3.0)	1.9 (1.2-3.1)	1.9 (1.2-3.1)	2.0 (1.2-3.2)	2.0 (1.2-3.1)	1.8 (1.1-3.0)	1.8 (1.1-3.0)	1.8 (1.1-2.9)
Major CVD**^*c*^**		1.5 (1.03-2.3)		1.5 (1.001-2.2)					1.5 (1.01-2.3)
New risk markers									
	MDRD (SD)	0.83 (0.68-1.01)			0.81 (0.65-3.7)				1.1 (0.85-1.3)
	CRP (SD)	1.3 (1.03-1.5)				1.3 (1.1-1.6)			1.2 (0.97-1.4)
	HCY**^*b*^** (SD)	1.4 (1.1-1.6)					1.3 (1.1-1.6)		1.2 (0.99-1.6)
	NT-proBNP (SD)	1.7 (1.4-2.1)						1.8 (1.4-2.2)	1.6 (1.3-2.1)

CRP, homocysteine and NT-proBNP are log transformed;***^a^*** including incident stroke, myocardial infarction or cardiovascular mortality;***^b^*** HCY: homocysteine;

*c* including a history of myocardial infarction, stroke or arterial surgery

Added individually to a multivariable model with all the traditional risk markers, the estimates showed no major change. In a multivariable analysis with all old and new markers, current smoking (HR 1.8 (95% CI 1.1-2.9)), a history of major CVD (HR 1.5 (95% CI 1.01-2.3)) and NT-proBNP (HR 1.6 (95% CI 1.3-2.1)), were still independently associated with an increased risk of cardiovascular morbidity/mortality. 

### C-statistics

The combination of traditional risk markers had a C-statistic of 0.59 (95% CI 0.52-0.66) (Table 3). Addition of NT-proBNP alone to all traditional risk markers increased the C-statistic to 0.67 (95% CI 0.61-0.74) with a p-value for ∆ C-statistic of 0.023 (Figure 1). 

**Table 3:** C- statistic, categoryless net reclassification improvement (NRI), integrated discrimination improvement (IDI) and relative IDI (rIDI) of the different models for prediction of five-year cardiovascular morbidity and mortality^***a***^ in participants with a history of cardiovascular disease (N=282), all compared to a model with traditional risk markers^***b***^.

	C-statistic	95% CI	∆ C-statistic**^*c*^**	p-value∆^c^	categoryless NRI**^*c*^** (%)	p-valueNRI**^*c*^**	IDI**^*c*^**	p-valueIDI**^*c*^**	rIDI**^*d*^**
Traditional risk markers	0.59	0.52-0.66							
+ major CVD	0.60	0.53-0.67	0.01	0.59	27.2	0.03	0.012	0.30	0.46
+ MDRD	0.59	0.52 -0.66	0.00	0.74	6.2	0.61	0.0025	0.81	0.095
+ CRP	0.60	0.54 -0.67	0.01	0.65	16.8	0.17	0.008	0.47	0.31
+ Homocysteine	0.62	0.55 -0.69	0.03	0.27	24.7	0.04	0.019	0.13	0.72
+ NT-proBNP	0.67	0.61 -0.74	0.08	0.023	39.0	0.001	0.067	<0.001	2.55
+ all five	0.69	0.62- 0.75	0.10	0.0067	50.8	<0.001	0.089	<0.001	3.39

CRP, homocysteine and NT-proBNP are log transformed;

*a* including incident stroke, myocardial infarction or cardiovascular mortality;

*b* including sex, systolic blood pressure, total cholesterol, HDL cholesterol, diabetes, current smoking;

*c* for comparison against traditional risk markers;

*d* for comparison with the discrimination of the model with the traditional risk markers (0.026 in this study)

### Categoryless net reclassification improvement (NRI)

For all different models we calculated the categoryless NRI, the net percentage of participants that is correctly reclassified when the new risk marker is added to the reference model with traditional risk markers (Table 3). Addition of a history of major CVD reclassified 27.2 % (p=0.03) of the participants correctly. For CRP this was 16.8 % (p=0.17), for homocysteine 24.7% (p=0.04) whereas for NT-proBNP it was 39.0 % (p=0.001). 

### Integrated discrimination improvement (IDI)

The discrimination of the baseline model, based on the predicted probabilities in those with and without events, was 0.026. The IDI after addition of a history of major CVD, or MDRD, CRP or homocysteine was not significant (Table 3). However, addition of NT-proBNP improved the discrimination to 0.067 (p<0.001). Thus for NT-proBNP the *relative* discrimination improvement, compared to the discrimination of the baseline model with traditional risk markers, was 2.55; this means that the discrimination of the model with addition of NT-proBNP is more than two and a half times as good as the discrimination using the model with the traditional risk markers. 

**Figure 2:**
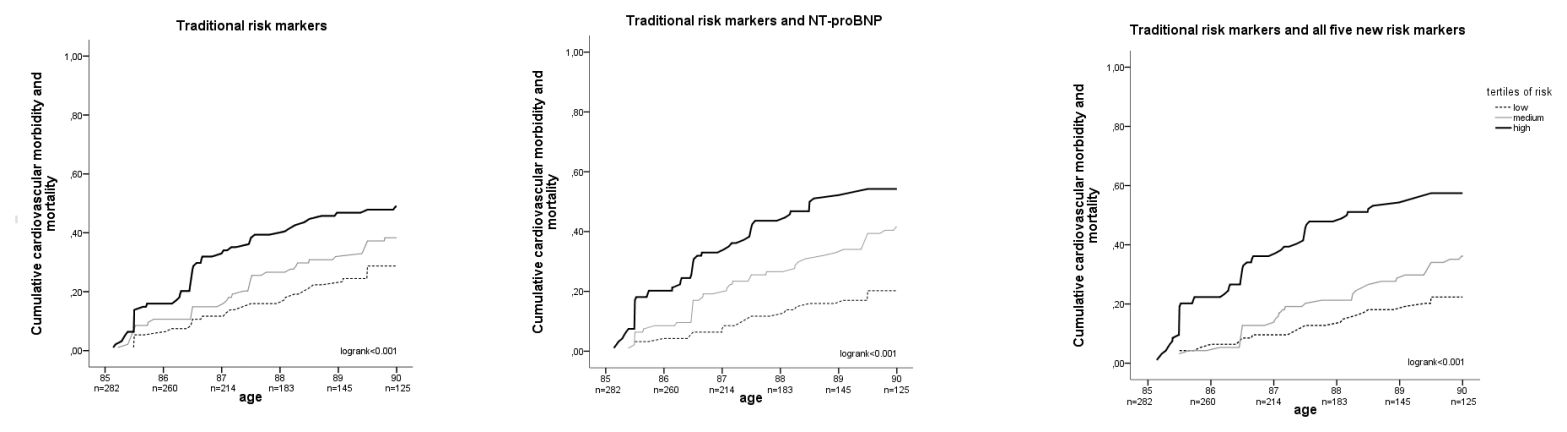
Kaplan-Meier curves, showing cumulative cardiovascular morbidity and mortality. Kaplan-Meier curves, adjusted for competing risks, showing cumulative cardiovascular morbidity and mortality for tertiles of risk for three different models: traditional risk markers only (left graph), traditional risk markers plus NT-proBNP (middle graph), and traditional risk markers plus all five new markers (a history of major cardiovascular disease, MDRD, CRP, homocysteine and NT-proBNP) (right graph) (N=282).

Figure 2 presents the Kaplan-Meier curves, adjusted for competing risks, for 5-year cumulative cardiovascular morbidity or mortality for the model with traditional risk markers, the model with NT-proBNP and the model with all five new markers.

When the analyses were repeated after exclusion of all participants with a history of heart failure according to information as obtained from their general practitioner or nursing home physician at baseline (n=68), results did not materially change (data not shown).

Cross validation of the model with the traditional risk markers led to a C-statistic of 0.53 (95%CI 0.46-0.60) for the traditional risk markers and a C-statistic of 0.64 (95%CI 0.58-0.71) for the model with addition of NT-proBNP. The difference between these two cross validated C-statistics was 0.11 (p=0.001). The cross validated model with addition of all five new markers had a C-statistic of 0.65 (95%CI 0.58-0.71), difference 0.12 (p=0.001).

## Discussion

In this study in very old subjects with established CVD, traditional risk markers had little predictive value for recurrent cardiovascular events and cardiovascular mortality. Of all biomarkers NT-proBNP was by far the strongest, adding substantial predictive value beyond the traditional risk markers. Besides NT-proBNP, the nature of the CVD history is also a relevant risk predictor: participants with a history of MI, stroke or arterial surgery had a higher recurrence rate than those with a less severe CVD history. Addition of information about the CVD history improved risk classification in about one in four participants. MDRD was not related to the endpoint, CRP was related but failed to show substantial incremental value, and homocysteine was both related to the endpoint and improved classification. However, NT-proBNP was the only risk marker that showed unambiguous improvement of prediction in all currently advocated methods of evaluation.

In comparison with the literature, in the present study, the traditional risk markers had little predictive value, with a C-statistic of 0.59, compared to 0.67 in secondary prevention in younger age groups [40]; this suggests that, in secondary prevention in very old age, traditional risk markers indeed loose predictive value, as they do in primary prevention [41].

Although we already knew that the nature of the CVD history had prognostic value in very old age [19], we now found it also has incremental value in prediction models in secondary prevention. 

New risk markers (renal markers (urinary albumin, eGFR, cystatin C) [31], CRP [30], homocysteine [25,42] and especially NT-proBNP [23,28,43–45] both individually and in various combinations have shown incremental predictive value in secondary prevention [27,46]. However, these studies mainly included younger participants. 

We have now demonstrated that in the oldest old with established CVD, NT-proBNP is the most potent predictor for recurrent cardiovascular morbidity and cardiovascular mortality. In line with the literature [26,28,47] CRP and homocysteine, although associated with the outcome, show little additional predictive value when NT-proBNP is also available. As described earlier in very old age [48], CRP was a weaker marker in our study than in younger age groups . In the present study MDRD was not predictive for recurrent cardiovascular morbidity and cardiovascular mortality. Cystatin C may be a superior risk marker of kidney function in very old age [49].

The present study has several strengths. The Leiden 85-plus Study is an observational study of a cohort of very old inhabitants of the city of Leiden with no exclusion criteria and a high participation rate. Therefore, our results can be applied to the general population of the oldest old. Also, the laboratory tests used are easy to obtain in daily practice and are often already available to the physician. A limitation is the relative small sample size of this cohort. We therefore recommend validation of our findings in a larger cohort. Another limitation is that, although addition of NT-proBNP was shown to improve the prediction of cardiovascular morbidity and cardiovascular mortality, we do not know whether incorporating this new risk marker in risk stratification in daily practice indeed improves CVD-free survival. Knowing that participants during the time of our study were not treated according to current guidelines, optimizing such treatment with statins and antihypertensive medication in participants with high NT-proBNP might considerably improve prognosis. 

Regarding clinical implications, we think that physicians may already improve their risk estimation by using available knowledge on patients’ CVD history: patients with a history of major CVD are at increased risk. Likewise, CRP and homocysteine are related to bad cardiovascular outcomes and may also be used when available. However, our results call for incorporation of NT-proBNP in risk estimation in secondary prevention in very old age as it improves identification of high-risk patients that will probably benefit most from intensified secondary preventive treatment and follow-up. 

In conclusion, the use of NT-proBNP should be considered when estimating risk for recurrent cardiovascular events and cardiovascular mortality in secondary prevention in very old age. 

## Novelty and Significance

**What Is New?**

• In secondary prevention in very old patients, measurement of NT-proBNP markedly improves prediction of recurrent cardiovascular events and cardiovascular mortality.

**What Is Relevant?**

• NT-proBNP might be used to select older people at the highest risk for recurrent cardiovascular events who may benefit most from strict secondary prevention.

**Summary**

Among very old subjects with established CVD, NT-proBNP was the strongest risk marker for recurrent cardiovascular events and cardiovascular mortality and considerably adds prognostic value on top of traditional risk markers. When estimating risk in secondary prevention in very old age, use of NT-proBNP should be considered.
